# A practical guide for the diagnosis of abdominal angiostrongyliasis caused by the nematode *Angiostrongylus costaricensis*

**DOI:** 10.1186/s13071-023-05757-6

**Published:** 2023-04-29

**Authors:** Rubens Rodriguez, Javier Mora, Alberto Solano-Barquero, Carlos Graeff-Teixeira, Alicia Rojas

**Affiliations:** 1 Laboratório de Anatomia Patológica e Citopatologia São Camilo-DASA, Maringá, Paraná Brazil; 2 Laboratory of Helminthology, Faculty of Microbiology, University of Costa Rica, San José, Costa Rica; 3 Centro de Investigación en Enfermedades Tropicales, University of Costa Rica, San José, Costa Rica; 4 Nucleo de Doenças Infecciosas, Centro de Ciências da Saúde, Universidade Federal do Espírito Santo, Vitória, Brazil

**Keywords:** *Angiostrongylus costaricensis*, Histopathology, Diagnostic guide, Infectious diseases

## Abstract

Abdominal angiostrongyliasis (AA) is a severe parasitic infection caused by the nematode *Angiostrongylus costaricensis*. This disease is characterized by abdominal pain, a strong inflammatory eosinophilic response in the blood and tissues, and eventually intestinal perforation. Diagnosis of AA is challenging since there are no commercially available serological kits for *A. costaricensis*, and thus, histopathological analysis remains the gold standard. Herein we provide a decision flowchart for clinicians to improve the diagnosis of AA based on a patient’s clinical manifestations, laboratory findings, macroscopic observations of the gut lesions, as well as characteristic microscopic alterations in biopsies. A brief discussion of the available polymerase chain reaction and in-house serological methods is also presented. The aim of this mini-review is to improve the diagnosis of AA, which should lead to prompt detection of cases and better estimates of the epidemiology and geographical distribution of *A. costaricensis*.

**Graphical Abstract:**

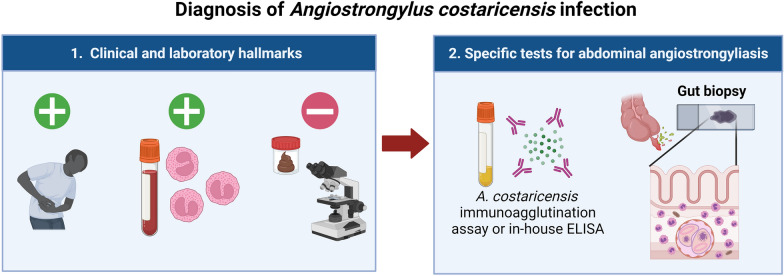

## Background

*Angiostrongylus costaricensis* (family Angiostrongylidae) is a parasitic nematode of rodents that can be transmitted to humans by the ingestion of infected slugs or snails and is the agent responsible for abdominal angiostrongyliasis (AA), a severe intestinal disease of humans [1, 2]. *Angiostrongylus costaricensis* is closely related to the rat lungworm *Angiostrongylus cantonensis* which induces eosinophilic meningoencephalitis in humans [3]. Specific environmental conditions are necessary for the completion of *A. costaricensis*’ life cycle, such as warm temperatures and high humidity, which are favored by the abundant rainfall of tropical and subtropical South and North America [4]. This parasite has been reported in 24 countries of the Americas and the Caribbean, from the Southern United States to Northern Argentina [5], in which all the abiotic conditions for the parasite’s development are met. In these geographical locations, the nematode has been reported to cause infections in humans, or has been detected in its natural intermediate or definitive hosts.

The life cycle of *A. costaricensis* involves slugs or snails as intermediate hosts [2] and rodents as definitive hosts. There have also been a few reports of this parasite in domestic dogs, and wild animals, such as raccoons, non-human primates, and opossums [2, 6–8]. Third-stage larvae present in fibromuscular tissues or slime of snails or slugs [1] are ingested by definitive hosts, in which they follow a complex lymphatic-venous-arterial pathway until they reach the mesenteric or ileocolic artery [9]. In this final niche, the worms develop into male and female adults which copulate; the eggs are released into the gut mucosa where they concomitantly hatch into first-stage larvae [10]. The latter are released in the feces of the definitive host and can infect intermediate hosts via oral or transdermal pathways to finally develop into third-stage larvae in fibromuscular tissues or mucous gland ducts [11, 12]. Humans are considered accidental dead-end hosts since the hatching of eggs in their gut mucosa and the shedding of stage-one larvae in their feces are impaired by a strong eosinophilic response [13].

**Fig. 1:**
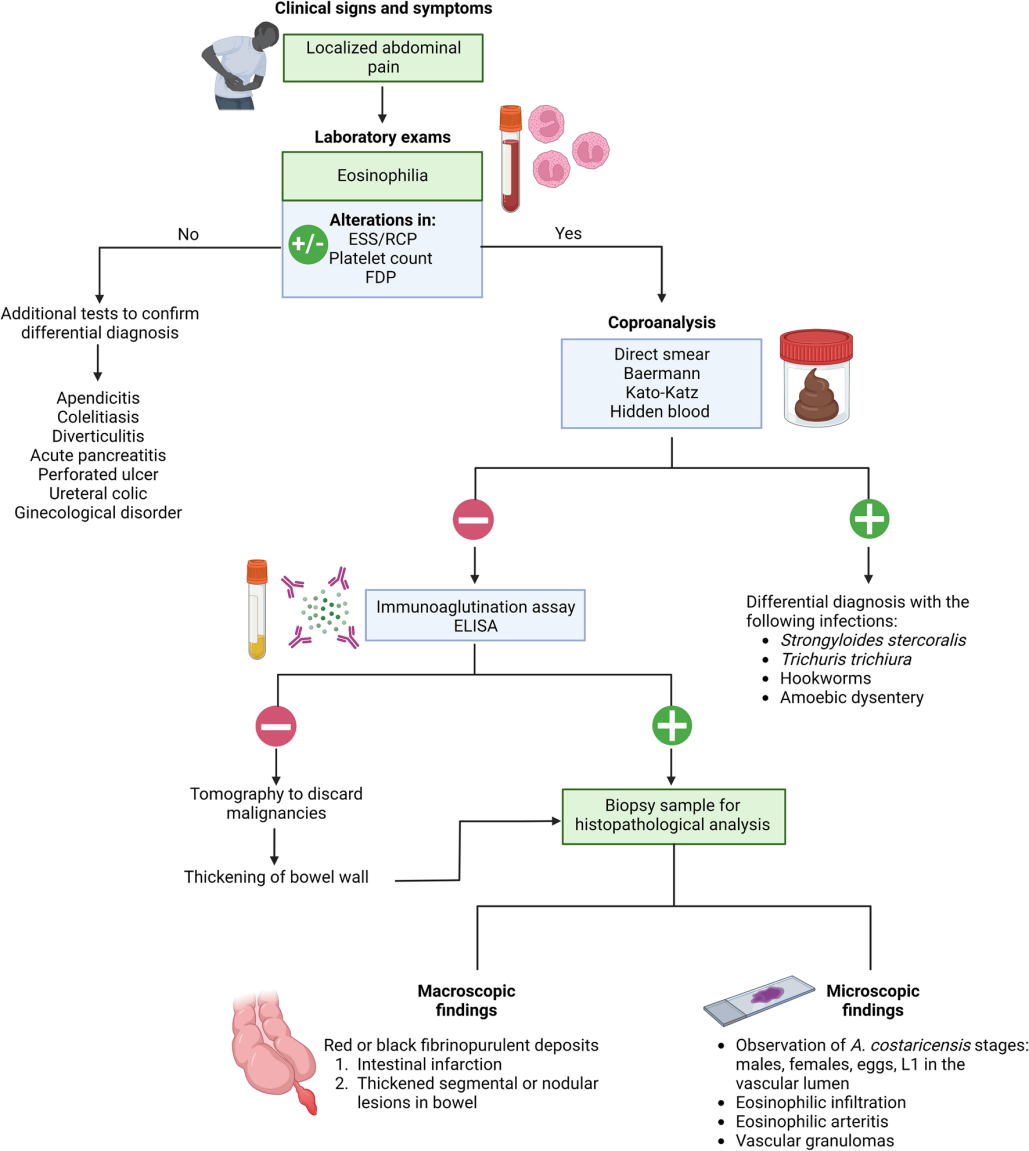
Recommended decision flowchart for the diagnosis of abdominal angiostrongyliasis (AA). When typical clinical manifestations of AA are first observed in patients, several laboratory tests should be performed, including measurement of inflammatory blood markers and a hemogram showing cell counts and leukocyte percentages. If no eosinophilia is found, other pathologies are suspected. However, if the patient has eosinophilia, a parasitic infection is presumed to be present. A complete coprological analysis should then be performed to discard the possibility that the infection is due to other gastrointestinal parasites. An immunoagglutination assay or enzyme-linked immunosorbent assay for *Angiostrongylus costaricensis* is recommended if no parasitic agent is found during coprological analysis. If the latter assays are negative, a computed tomography scan is recommended to rule out possible gut malignancies. However, if the enzyme-linked immunosorbent assay or immunoagglutination tests are positive, a biopsy of the affected gut section should be analyzed to confirm infection by *A. costaricensis*. Key macroscopic and microscopic alterations of the gut tissue infected with this parasite are indicated. *ESS* Erythrocyte sedimentation speed, *RCP* reactive C-protein, *FDP* fibrinogen-derived products. This figure was created using BioRender.com

AA is mainly reported in school-age children and young adults [2], and is characterized by abdominal pain and blood/tissue eosinophilia, resulting from a severe inflammatory reaction, and eventually intestinal perforation [14–16]. These clinical manifestations are explained by the presence of adult worms in arteries and the strong eosinophilic response induced in situ. Extraintestinal complications are less frequently reported, but may include nodular hepatic lesions [17] or testicular necrosis [18]. In this guide, we propose a diagnostic flowchart to aid clinicians in the diagnosis of AA (Fig. 1), starting with the identification of clinical manifestations in patients, followed by the general as well as *A. costaricensis-*specific laboratory tests that need to be performed, and concluding with the main histopathological findings on biopsies.

## Diagnosis of AA

Diagnosis of AA is generally confirmed by the identification of *A. costaricensis* eggs, larvae or adult worms during histopathological analysis of the vermiform appendix and small and large bowel [19], although rare cases of testicular and liver disease have also been reported [20–22]. It is also possible to confirm the diagnosis in suspected cases by polymerase chain reaction (PCR) using DNA from formalin-fixed paraffin-embedded tissues (FFPE) [23]. Nucleic acid detection in serum has also been standardized for AA using primers targeting *A. cantonensis* sequences [24, 25]. Studies are underway to design additional assays using *A. costaricensis* sequences.

### Histopathology: macroscopic and microscopic findings

**Fig. 2a–f:**
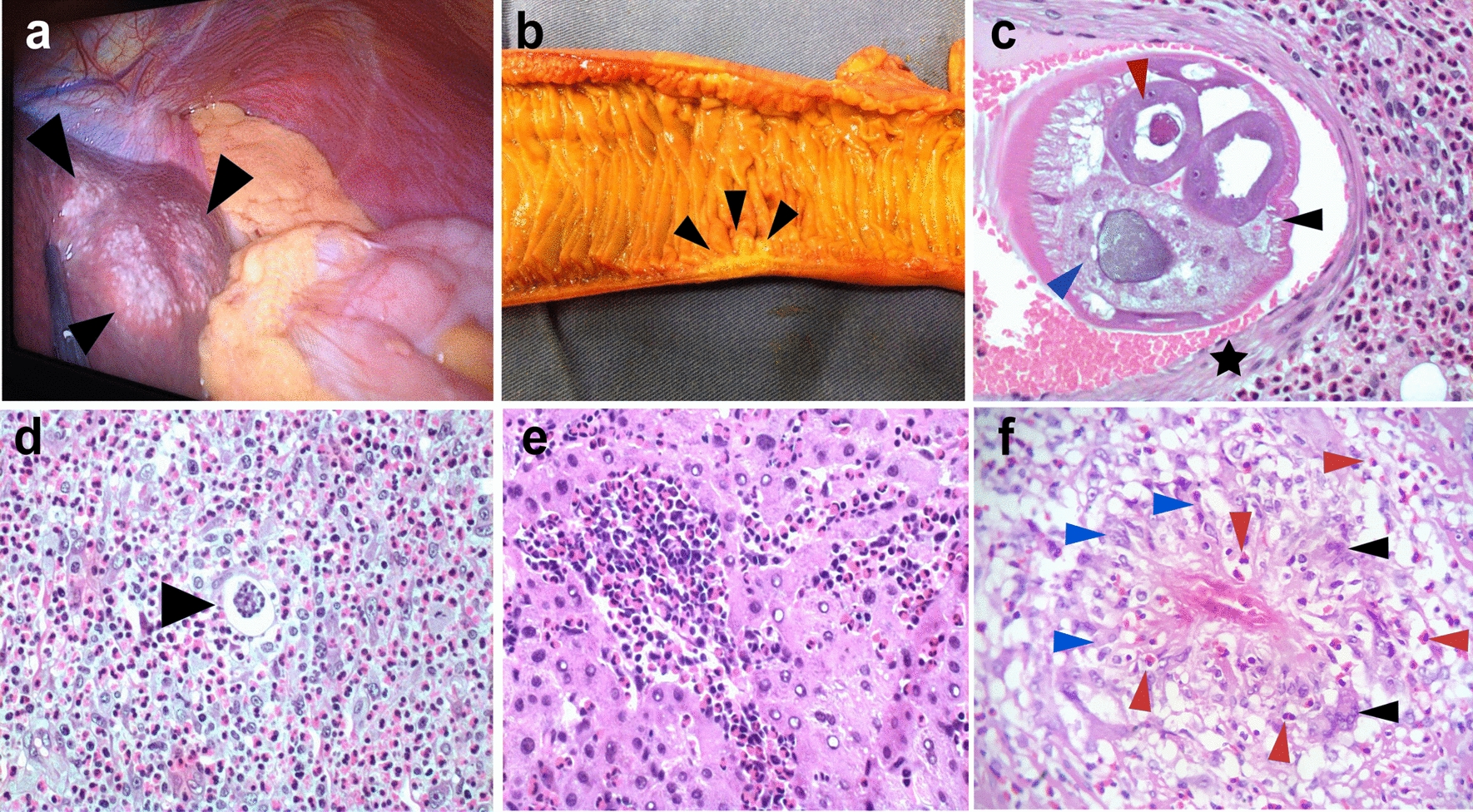
Macroscopic and microscopic histopathological findings of AA. **a** Extraintestinal angiostrongyliasis in liver with multiple small nodules (black arrowheads) and yellowish material. **b** Small bowel showing a segmental Crohn disease-like lesion with wall thickening and hemorrhagic area (black arrowheads). **c** Transversal section of *Angiostrongylus costaricensis* adult female worm inside a branch of the mesenteric artery showing polymyarian musculature (black arrowhead), uterus with an egg inside (red arrowhead) and gut (blue arrowhead). Muscle cells of the artery are shown by a black star [hematoxylin–eosin staining (HE), ×200]. **d** *Angiostrongylus costaricensis* egg (black arrowhead) inside a small vessel surrounded by severe eosinophilic infiltration (HE, ×400). **e** Eosinophilic infiltration in the liver of a patient with AA (HE, ×400). **f** Granuloma with histiocytes (blue arrowheads), giant multinucleated cells (black arrowhead) and eosinophils (red arrowheads) (HE, ×400)

Macroscopically, an appendix infected with *A. costaricensis* is similar in appearance to that seen in routine cases of acute appendicitis, with red or black fibrinopurulent deposits in the serosa and a thickened wall (Fig. 2a, b) [13]. To increase the chances of finding parasitic structures during the microscopic analysis, when preparing the FFPE it is necessary to include the entire vermiform appendix and the mesoappendix [23].

Two types of macroscopic lesions are observed in the bowel: intestinal infarction, and segmental and nodular lesions in the large bowel with thickened areas. There may be multiple segmental and nodular lesions, which may resemble Crohn disease [26] (Fig. 2b). It is important to perform adequate sampling of the infarcted lesions by randomly selecting areas of the intestinal wall and undertaking extensive sectioning of the mesentery. If the diagnosis is not confirmed from the first sampling, it is necessary to embed the entire surgical specimen in paraffin since parasitic structures may be absent from some sections. Moreover, blocks of the entire macroscopic lesion are required when sampling segmental or nodular lesions. In these cases, samples of the mesentery should be taken only from the hemorrhagic sites or thickened vessels [23]. It is crucial to carefully analyze the antimesenteric portion of the bowel, where eggs or larvae may be found in capillary lumens [13].

Routine hematoxylin and eosin staining is used for microscopic analysis of AA. The diagnosis of AA is confirmed when eggs, larvae or sections of adult worms are found in the lumens of capillaries, arterioles and large arteries [5, 23]. Adult worms are found mainly in submucosal, muscular, serosa or large mesenteric/mesoappendix arteries, where they may be associated with thrombosis and infarction (Fig. 2c) [27, 28]. In our experience, the majority of adult worms are found in the submucosal and mesenteric arteries.

A type 2 inflammatory response induced by *A. costaricensis* generates a strong eosinophilic infiltration [5], a key observation during the microscopic examination of biopsies (Fig. 2c–f). Eosinophilic infiltration around capillaries and arterioles of the submucosa and muscularis propria is associated with severe disease. Eosinophilic infiltration of arterial walls, which is termed eosinophilic arteritis, is a histopathological feature of AA (Fig. 2c) [26]. Additionally, granulomas are observed in the walls of large arteries [26] or engulfing capillaries and arterioles, together with the presence of eggs or larva in the lumen [5, 29]. Severe granulomatous reactions in the submucosa and muscularis propria of the large bowel leads to pseudotumor formation together with small vessel occlusion due to inflammation. Necrosis of the mucosa or intestinal wall and secondary ulceration may occur in severe disease [29].

AA is associated with characteristic clinical manifestations in patients, the observation of eosinophilic infiltration, eosinophilic arteritis and granulomas engulfing capillaries and arterioles, as well as key epidemiological features. Sometimes it is necessary to include the complete surgical specimen in FFPE blocks and analyze serial slides of the suspected infected areas to identify parasitic structures.

### In-house serological methods

In-house serological assays like a latex agglutination test (Morera test) and an immunoglobulin G (IgG)–enzyme-linked immunosorbent assay are available in Costa Rica (from the Instituto Costarricense de Investigación y Enseñanza en Nutrición y Salud, Cartago) and Brazil (from the Instituto Adolfo Lutz, São Paulo), and both use whole somatic *A. costaricensis* antigens [30]. Crude antigens from eggs have been evaluated for serological assays [31] but are not used in routine diagnostic tests.

Difficulties in obtaining large numbers of *A. costaricensis* adult specimens and maintaining the parasite’s life cycle have prompted the use of whole crude antigens [32] and recombinant proteins (galectin) [33] from its congeneric species *A. cantonensis* for serological testing [32], which have proven successful. Since these assays use heterologous proteins of *A. cantonensis*, epidemiological factors as well as the patient’s clinical history and manifestations should be considered for the correct interpretation of the results and to discard infection with *A. cantonensis*. It is highly likely that future protocols will involve rapid tests that use homologous recombinant antigens [33, 34]. Besides improving the reproducibility of these assays, highly purified and well-characterized *A. costaricensis* antigens may prevent cross-reactivity with other nematode species, and specifically to the threadworm *Strongyloides stercoralis* [31, 35]. Increased specificity may also result from the detection of IgG1 antibodies, as suggested by Abrahams-Sandi et al. [31]. The reactivity of human IgG decreases with time in post-acute infections, but it may remain detectable for several months [36].

### PCR methods

Three DNA-based methods have been designed for confirming the diagnosis of AA. The first method was a conventional end-point PCR which used a 232-base pair fragment of a 66-kDa muscle protein of female *A. cantonensis* (Ac-fmp-1) as a target. This reaction detected the Ac-fmp-1 homologue in *A. costaricensis* [24] in the sera of two out of three patients with AA. Moreover, the PCR did not cross-amplify DNA of other gastrointestinal nematodes such as *Strongyloides ratti*, *Ancylostoma caninum*, *Ascaris suum*, and *Toxocara canis* [24].

The second conventional end-point PCR method used FFPE samples from patients with confirmed AA to detect the same Ac-fmp-1 homologous DNA fragment of *A. costaricensis* [23]. This method detected 55% (11/20) of cases confirmed by histopathology, especially in sections containing parasitic structures or granulomas [23]. Overall, this PCR showed intermediate sensitivity and high specificity, since the reactions were negative for FFPE samples of negative controls and FFPE samples with *Ascaris lumbricoides*, *Enterobius vermicularis*, *Strongyloides stercoralis* and *Schistosoma mansoni* [23].

The third molecular assay was a real-time PCR that also amplified a DNA fragment of the Ac-fmp-1 of *A. costaricensis* in sera of patients with presumptive AA [25]. This real-time PCR detected the parasite’s DNA in two out of 28 sera matched to patients with AA. In addition, the two samples positive in the real-time PCR were negative according to an indirect enzyme-linked immunosorbent assay. Therefore, the method confirmed the presence of the nematode’s DNA in sera of patients with suspected AA and complemented the results of serological techniques, suggesting that the assay might be useful during the acute phase of the infection [25].

## Conclusions

An interdisciplinary approach is needed to solve the current challenges in the diagnosis of AA. This approach includes education as well as regularly updating healthcare professionals and pathologists about the clinical characteristics of this parasitosis, which in turn should promote awareness of potential AA cases in geographic regions where the infection has not been reported before. Finally, the histopathological criteria summarized here should be adequate for the diagnosis of AA, and may help us better understand the epidemiology and distribution of this parasite. However, further studies are required to develop a sensitive and specific molecular diagnostic tool for AA to improve the quality of the clinical approach for patients with this disease by reducing the amount of time lost prior to diagnosis and avoiding the risks that are associated with biopsy.

## Data Availability

Not applicable.

## Abbreviations

AA: Abdominal angiostrongyliasis
FFPE: Formalin-fixed paraffin embedded
